# How to increase COVID-19 vaccine uptake among children? determinants associated with vaccine compliance

**DOI:** 10.3389/fped.2022.1038308

**Published:** 2023-01-13

**Authors:** Moshe Hoshen, Vered Shkalim Zemer, Shai Ashkenazi, Zachi Grossman, Maya Gerstein, Noga Yosef, Moriya Cohen, Herman Avner Cohen

**Affiliations:** ^1^ Dan-Petach Tikva District, Clalit Health Services, Petach Tikva, Israel; ^2^ Bioinformatics Department, Jerusalem College of Technology, Jerusalem, Israel; ^3^ Sackler Faculty of Medicine, Tel-Aviv University, Tel Aviv, Israel; ^4^ Adelson School of Medicine, Ariel University, Ariel, Israel; ^5^ Maccabi Healthcare Services, Tel Aviv, Israel; ^6^ Pediatric Ambulatory Community Clinic, Petach Tikva, Israel; ^7^ Microbiology Unit, Ariel University, Ariel, Israel

**Keywords:** coronavirus-2019, COVID-19 vaccine, children, co-morbidity, vaccine hesitancy, parental vaccination

## Abstract

**Objective:**

Three aims: to elucidate determinants associated with COVID-19 vaccine uptake in children and the association with parental vaccination; to compare rates of PCR-positive SARS-CoV-2 results between vaccinated and unvaccinated children; to estimate the rate of parental COVID-19 vaccination and its association with the vaccination rate of their children.

**Methods:**

We performed a retrospective chart review of all children aged 5–11 years registered at a central district in Israel from November 21st, 2021 to April 30th, 2022, and characterized COVID-19 vaccinated vs. unvaccinated individuals. Data retrieved from the electronic medical files included: demographics [age, gender, sector, socioeconomic status (SES)]; COVID-19 vaccination (first and second doses) and influenza vaccination status; co-morbidities; and parental vaccinations for COVID-19. We divided the population into three distinct demographic groups: non-ultra-orthodox Jews (43,889 children), ultra-orthodox Jews (13,858 children), and Arabs (4,029 children).

**Results:**

Of the 61,776 children included in the study, 20,355 (32.9%) received at least one dose of the COVID-19 vaccine. Vaccination rates were similar amongst males and females and were higher in children aged 9–11 years compared to children aged 5–6 years. Multivariate analysis identified five independent determinants that were significantly (*p *< 0.001) associated with low vaccination rates: Arab and ultra-orthodox sectors (odds ratios: 0.235 and 0.617, respectively); children aged 5–8 years; children of low SES; and children who had not received previous seasonal influenza vaccination. Relatively high vaccination rates were noted amongst children with the following medical co-morbidities: treatment with biological agents (42.9%); solid tumor transplantation (42.9%); type 1 diabetes mellitus (38.5%), asthma (38.2%), and attention deficit and hyperactivity disorder (ADHD) (37.6%). Regarding the uptake of two vaccine doses among children with co-morbidities, it was highest in those with type 1 diabetes mellitus, heart failure, treatment with biological agents, asthma and obesity.

**Conclusion:**

This study highlights several pediatric sub-populations with low and high vaccine uptake. It is essential to focus on determinants associated with low vaccination rates.

## Introduction

The coronavirus disease-19 (COVID-19) pandemic caused enormous health, economic and societal burdens worldwide (1). As of July 25, 2022, the World Health Organization (WHO) recorded 566,977,818 confirmed cases, including more than 6.3 million deaths, and as of July 18, 2022, a total of more than 12.2 billion vaccine doses have been administered (2). Children have a milder course of acute COVID-19 compared to adults (3, 4). The prevalence of severe acute respiratory syndrome coronavirus-2 (SARS-CoV-2) infection is lower in the pediatric population, with lower mortality rates (5, 6). However, children can also present with a severe course and develop complications, such as pediatric inflammatory multisystem syndrome (PIMS) and long-COVID (7–9). Children with underlying medical conditions such as obesity, diabetes, heart disease, chronic lung diseases other than asthma, seizure disorders, and immunosuppression were reported to have a higher prevalence of severe COVID-19 (10). In addition, children may be more susceptible to the negative mental health effects of the COVID-19 pandemic and the response measures (11). Samji et al. noted that children and adolescents experienced more depressive and anxious symptoms compared to the pre-pandemic rates, specifically with high levels of fear and concern regarding the impact of COVID-19 on their lives (11).

COVID-19 vaccines have had a crucial role in decreasing SARS-CoV-2 infection rates and COVID-19 disease. Vaccination has a double effect: direct protection of vaccinated individuals and indirect protection of individuals living in vaccinated environments (12). Several studies reported that a substantial amount of COVID-19 transmission occurs in close, crowded, and prolonged contact, such as in households (13–16). These household studies may be alternatives to larger populations for estimating the direct and indirect effects of vaccines (12).

The COVID-19 vaccine produced by Pfizer-BioNTech (BNT162b2) was authorized in Israel in December 2020 for individuals aged 16 years and older. In May 2021, the authorization was extended to children and adolescents aged 12 years and older, and in November 2021 – to children aged 5 years and older (17).

The Israeli population accounts for more than 9 million people: approximately 74% are Jews, 21% are Arabs and 5% are of other ethnicities. About 12% of the Jewish population belongs to a distinct subpopulation that is religiously ultra-orthodox. In our study, we divided the population into three distinct demographic groups, which we referred to as “sectors”: non-ultra-orthodox Jews, ultra-orthodox Jews, and Arabs. These sectors differ in their cultural and social characteristics. The Arab and ultra-orthodox Jewish populations have high fertility rates, are younger compared to the non-ultra-orthodox Jewish population, and tend to live in close communities with limited access to the general media (18, 19). In general, the ultra-orthodox Jewish community is a traditional society, with little contact with the general population (by choice). It has limited income, includes large families crowded in small apartments in overpopulated neighborhoods, and has strong community bonds In the ultra-orthodox Jewish population the Rabbi, the spiritual leader, plays a central role in community actions and behaviors. Healthcare decision-making among the ultraorthodox generally involves seeking opinions from rabbinical leaders, who sometimes rule in opposition to the physician's opinion. In addition, ultra-orthodox are less likely to work in the field of health (18, 19).

The primary aim of our study was to illuminate factors that are associated with the acceptance of the COVID-19 vaccine in children aged 5–11 years by comparing vaccinated and unvaccinated individuals. The secondary aim was to compare the rates of PCR-positive SARS-CoV-2 results between vaccinated and unvaccinated children. The third aim was to estimate the rate of parental vaccination with the COVID-19 vaccine and its association with the vaccination rate of their children.

## Materials and methods

### Setting and data source

Clalit Health Services (CHS) is the largest Israeli health fund. It comprises a comprehensive computerized database, which is continuously updated with regard to subjects' demographics, community and outpatient visits, laboratory tests, hospitalizations, medication prescriptions, and purchases. During each physician visit, a diagnosis is established according to the International Classification of Diseases, ninth revision (ICD-9).

### Study population

The study population consisted of all children aged 5–11 years who were registered in CHS in the Dan-Petach Tikva district during the study period of November 21st, 2021–April 30th, 2022. The beginning date of our study period was chosen according to the date when Israeli children aged 5–11 years started to receive the COVID-19 vaccine. The Dan-Petach Tikva district comprises about 500,000 members and includes large towns of mainly secular Jews, large towns of ultra-orthodox Jews, and a few Arab towns.

We divided the study population into two groups: unvaccinated and vaccinated with at least one dose of the vaccine, with further subgrouping according to the number of COVID-19 vaccine doses received. Data retrieved from the electronic database included: demographics (age, gender, sector, and socioeconomic status (SES), which was defined according to the classification of the Israeli Central Bureau of Statistics (20); household size; vaccinations for COVID-19 (first and second doses) and for influenza; co-morbidities; and parental vaccinations for COVID-19. Exclusion criteria were children who had had a positive result of PCR-COVID-19 test prior to the vaccination period in this age group.

The study was approved by the Clalit Community Institutional Review Board.

### Statistical analysis

We used descriptive statistics to report the demographic and clinical determinants of the vaccinated and unvaccinated study groups. Proportions were compared by *χ*^2^ or Fisher exact test, as appropriate, and continuous determinants by Student *t*-test or Mann–Whitney, as appropriate. We performed logistic regression to analyze the adjusted odds ratio of vaccination as the dependent determinant, based on known predictors of vaccination (age, gender, sector, SES, past influenza vaccination, co-morbidities, and parental vaccination). We conducted a univariate analysis to identify determinants associated with vaccine acceptance. Based on determinants found significant in the univariate analysis, we performed multivariate logistic regression to analyze the adjusted odds ratio of vaccination as the dependent determinant. We drew vaccination uptake curves by sector adjusting for age and gender. Analysis was performed with The R Project for Statistical Computing software (versions 4.1.0), using “survival” and “survminer” packages (21).

## Results

Of the 61,776 children in the study, 31,635 (51.2%) were males and 30,141 (48.8%) were females; 57,747 (93.5%) were Jews and 4,029 (6.5%), were Arabs.

### Determinants associated with COVID-19 vaccination rates

Table 1 presents the characteristics of the study population by COVID-19 vaccination. COVID-19 vaccination rates were similar among males and females (51.2% and 48.8%). Non-ultra-orthodox Jewish children had a much higher vaccination rate than either Arabs or ultra-orthodox Jews (41.2%, 13.4%, and 12.6%, respectively; *p* < 0.0001). Children with high SES had significantly higher vaccination rates than those with low SES (50.5% vs. 12.1%). Among 20,355 children who received at least one dose of the COVID-19 vaccine, 71.9% had received previous seasonal influenza vaccination. We noted that the highest vaccination rate was among households of four members each and the lowest was among families that included six persons or more (*p* < 0.0001).

**Table 1:** Study group variables associated with vaccination rates against COVID-19 (≥1 dose).

Characteristics	Study group			* *
	Total	Vaccinated	Unvaccinated	*p*-value
Children, no.	61,776	20,355 (32.9%)	41,421 (67.1%)	
**Age (years), mean (±SD)**	8.1 (1.9)	8.3 (1.9)	8.0 (1.9)	<0.001
Age group (years)				
5–6	15,621	4,241 (27.1%)	11,380 (72.9%)	<0.001
7–8	19,450	6,413 (33.0%)	13,037 (67.0%)	
9–11	26,705	9,701 (36.3%)	17,004 (63.7%)	
Gender				
Male	31,635	10,586 (33.5%)	21,049 (66.5%)	0.006
Female	30,141	9,769 (32.4%)	20,372 (67.6%)	
Sector				
Non-ultra-orthodox Jews	43,889	18,069 (41.2%)	25,820 (58.8%)	<0.01
Ultra-orthodox Jews	13,858	1,748 (12.6%)	12,110 (87.4%)	
Arabs	4,029	538 (13.4%)	3,491 (86.6%)	
Socioeconomic status				
Low	16,275	1,965 (12.1%)	14,310 (87.9%)	<0.001
Middle	20,098	5,565 (27.7%)	14,533 (72.3%)	
High	25,403	12,825 (50.5%)	12,578 (49.5%)	
Household number				
≤3	9,489	3,726 (39.3%)	5,763 (60.7%)	<0.001
4	14,764	5,992 (40.6%)	8,772 (59.4%)	
5	17,444	6,350 (36.4%)	11,094 (63.6%)	
≥6	20,079	4,287 (21.4%)	15,792 (78.6%)	
Underlying conditions				
Obesity	7,236	2,563 (35.4%)	4,673 (64.6%)	0.093
Type 1 diabetes	65	25 (38.5%)	40 (61.5%)	0.416
Asthma	1,176	449 (38.2%)	727 (61.8%)	<0.001
Epilepsy/seizures	326	120 (36.8%)	206 (63.2%)	0.140
Autism	67	23 (34.3%)	44 (65.7%)	0.140
ADHD	4,073	1,530 (37.6%)	2,543 (62.4%)	<0.001
Down syndrome	69	12 (17.4%)	57 (82.6%)	0.005
Heart failure	23	6 (26.1%)	17 (73.9%)	0.658
Chronic renal failure	22	5 (22.7%)	17 (77.3%)	0.428
Malignancy	117	36 (30.8%)	81 (69.2%)	0.686
Steroid therapy^a^	36	13 (36.1%)	23 (63.9%)	0.724
Biological therapy^b^	28	12 (42.9%)	16 (57.1%)	0.314
Solid tumor transplantation	7	3 (42.9%)	4 (57.1%)	0.876
BMT	13	2 (15.4%)	11 (84.6%)	0.293
**Influenza vaccination**^c^	32,221	14,635 (45.5%)	17,586 (54.6%)	<0.001

All variables are expressed as *n* (%) unless otherwise stated.

ADHD, attention-deficit hyperactivity disorder; BMT, bone marrow transplant.

^a^ Including prednisone or prednisolone at a dosage of ≥20 mg/day for at least two week.

^b^ Including alemtuzumab, adalimumab, certolizumab, infliximab, etanercept, rituximab, and anakinra.

^c^ In the previous 3 years.

Relatively high vaccination rates were noted amongst children with the following medical co-morbidities: treatment with biological agents (including alemtuzumab, adalimumab, certolizumab, infliximab, etanercept, rituximab, and anakinra) (42.9%); solid tumor transplantation (42.9%); type 1 diabetes mellitus (38.5%), asthma (38.2%), and attention deficit and hyperactivity disorder (ADHD) (37.6%). Table 2 presents the number of vaccine doses given to children with various co-morbidities. The highest second COVID-19 vaccine dose uptake was among children with type 1 diabetes mellitus, heart failure, treatment with biological agents, asthma, and obesity.

**Table 2:** Characteristics of the study group by number of COVID-19 vaccine doses received.

	Total no. of vaccinated children	Received 1st vaccine dose	Received 2nd vaccine dose	*p-value*
**Children no.**	20,355	5,809 (28.5%)	14,546 (71.5%)	
**Age (years), mean (±SD)**	8.1 (1.9)	8.1 (1.9)	8.3 (1.9)	
Age group (years)				
5–6	4,241	1,105 (26.1%)	3,136 (73.9%)	<0.0001
7–8	6,413	1,892 (29.5%)	4,521 (70.5%)	
9–11	9,701	2,812 (29.0%)	6,889 (71.0%)	
Gender				
Male	10,586	3,018 (28.5%)	7,568 (71.5%)	<0.0001
Female	9,769	2,791 (28.6%)	6,978 (71.4%)	
Sector				
Non-ultra-orthodox Jews	18,069	4,433 (24.5%)	13,636 (75.5%)	<0.0001
Ultra-orthodox Jews	1,748	1,029 (58.9%)	719 (41.1%)	
Arabs	538	347 (64.5%)	191 (35.5%)	
Socioeconomic status				
Low	1,965	1,094 (55.7%)	871 (44.3%)	<0.0001
Middle	5,565	1,816 (32.6%)	3,794 (67.4%)	
High	12,825	2,899 (22.6%)	9,926 (77.4%)	
Household number				
≤3	3,726	2,769 (74.3%)	957 (25.7%)	<0.0001
4	5,992	4,472 (74.6%)	1,520 (25.4%)	
5	6,350	4,662 (73.4%)	1,688 (26.6%)	
≥6	4,287	2,643 (61.7%)	1,644 (38.3%)	
Underlying conditions				
Obesity	2,563	725 (28.3%)	1,838 (71.7%)	0.565
Type 1 diabetes	25	4 (16.0%)	21 (84.0%)	0.243
Asthma	449	114 (25.4%)	335 (74.6%)	0.150
Epilepsy/seizures	120	52 (43.3%)	68 (56.7%)	0.0005
Autism	23	13 (56.5%)	10 (43.5%)	<0.0001
ADHD	1,530	451 (29.5%)	1,079 (70.5%)	0.415
Down syndrome	12	7 (58.3%)	5 (41.7%)	0.047
Heart failure	6	1 (16.7%)	5 (83.3%)	1
Chronic renal failure	5	2 (40.0%)	3 (60.0%)	0.942
Malignancy	36	11 (30.6%)	25 (69.4%)	0.933
Steroid therapy^a^	13	4 (30.8%)	9 (69.2%)	0.769
Biological therapy^b^	12	3 (25.0%)	9 (75.0%)	1
Solid tumor transplantation	3	1 (33.3%)	2 (66.7%)	1
BMT	2	1 (50.0%)	1 (50.0%)	1
**Influenza vaccination**^c^	14,635	3,871 (26.5%)	10,764 (73.5%)	<0.0001

All variables are expressed as *n* (%), unless otherwise stated.

ADHD, attention-deficit hyperactivity disorder; BMT, bone marrow transplant.

^a^ Including prednisone or prednisolone at a dosage of ≥20 mg/day for at least two week.

^b^ Including alemtuzumab, adalimumab, certolizumab, infliximab, etanercept, rituximab and anakinra.

^c^ In the previous 3 years.

**Figure 1:**
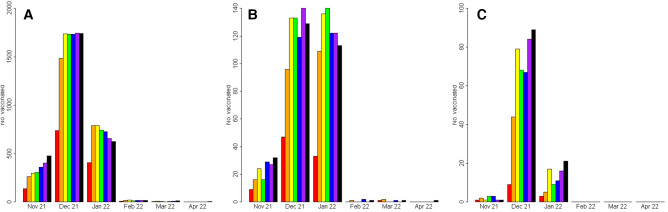
Timing of vaccination by sector and age. (**A**) Non-ultraorthodox Jewish children (**B**) Ultra-orthodox Jewish children (**C**) Arab children. Red- children aged 5 years; Orange- children aged 6 years Yellow- children aged 7 years; Green- children aged 8 years Blue- children aged 9 years; Violet- children aged 10 years Black- children aged 11 years.

Figure 1 depicts the monthly number of children vaccinated according to age group and sector (non-ultra-orthodox Jews, ultra-orthodox Jews, and Arabs). The Figure refers to the time period from vaccination approval for children by the Israeli Ministry of Health until April 30th, 2022. Overall COVID-19 vaccination uptake among children 5–11 years of age was higher amongst children aged 9–11 years compared to children aged 5–6 years. Regarding vaccination by sectorial distribution, the vaccination uptake was the highest among non-ultra-orthodox Jews, relatively low in the ultra-Orthodox Jews, and lowest among the Arab population.

Table 2 presents the characteristics of the study group by the number of vaccine doses received. The rates of acceptance of the first and second COVID-19 vaccine doses were very similar between females and males. Higher rates of two vaccine dose uptake were noted in the non-ultra-orthodox Jewish children, compared to children of ultra-orthodox Jews and Arabs. A higher number of two vaccine doses was given to children with high SES, compared to low SES, and to children belonging to a household size of 6 family members or more, compared to smaller household sizes.

### Vaccination outcomes

A total of 34,054 children were PCR-positive for SARS-CoV-2 during the study period. Of whom, 30.6% had been vaccinated against COVID-19. PCR-positivity was noted in 3,937 of 5,809 (67.8%) of those who had received a single vaccine dose as opposed to 6,473 of 14,546 (44.5%) of those who had received two vaccine doses. One child who died from COVID-19 illness was unvaccinated.

### Multivariate analysis

Table 3 presents the results of the multivariable logistic regression analysis of the factors associated with children's COVID-19 vaccination. Multivariate analysis identified five independent determinants that were significantly associated with low vaccination rates (*p* < 0.001): the Arab population and the ultra-orthodox Jewish sector; children aged 9–11 years (compared to children aged 5–6 years); children of low SES; and children who had not received previous seasonal influenza vaccination.

**Table 3:** Multivariable regression for the likelihood of COVID-19 vaccination among children aged 5–11 years.

Variable	Odds ratio	Lower 95% CI	Upper 95% CI	*p-value*
Arab	0.235	0.212	0.26	<0.0001
Ultra-orthodox Jew	0.617	0.566	0.672	
Age	1.061	1.048	1.047	
SES	2.364	2.237	2.458	
Influenza vaccination	3.921	3.572	4.305	

SES, socioeconomic status.

### Parental vaccination status

**Figure 2:**
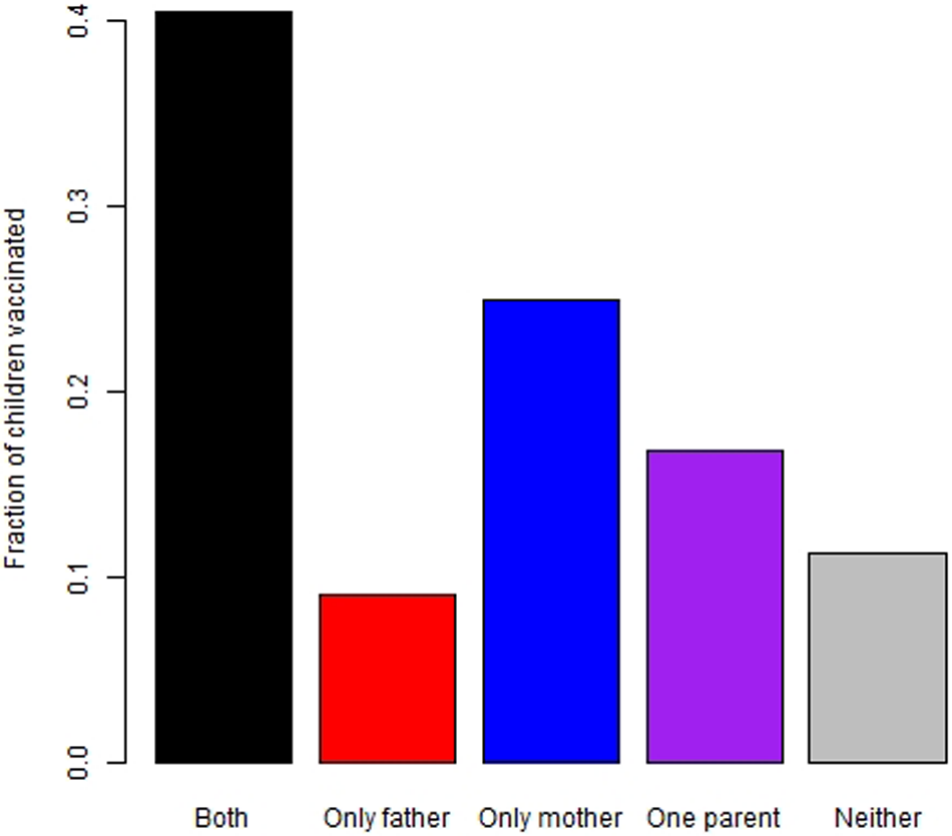
Children's vaccination rates by parental vaccination status black – both parents vaccinated; Red – only father vaccinated; blue – only mother vaccinated; violet – one parent vaccinated; grey- neither parent vaccinated.

Table 4 shows the parental vaccination status against COVID-19 and its association with their children's vaccination. The vaccination rate in children of families in which both parents had COVID-19 vaccination was highest (41%), compared to the vaccination rate in children of families in which only the mother or the father was vaccinated or neither parents were vaccinated (25%, 9%, and 11%, respectively). Figure 2 shows children's vaccination rate according to the status of parental vaccination.

**Table 4:** Parental vaccination status.

Parental vaccination	N	Study group children		
		Vaccinated	Not vaccinated	*p-value*
Total	72,082	22,088 (31%)	49,994 (69%)	<0.0001
Both parents vaccinated	45,793 (64%)	18,553 (41%)	27,260 (60%)	
Neither parents vaccinated	15,963 (22%)	1,802 (11%)	14,161 (89%)	
Only father vaccinated	5,247 (7%)	475 (9%)	4,772 (91%)	
Only mother vaccinated	5,059 (7%)	1,258 (25%)	3,801 (75%)	

All variables are expressed as *n* (%).

## Discussion

The present study illuminates determinants associated with COVID-19 vaccination among children aged 5–11 years and its association with parental vaccination. Overall, COVID-19 vaccination uptake among children 9–11 years of age was higher than among children aged 5–6 years. The reasons for the differences in vaccination coverage between older and younger children might reflect variations in parental hesitancy based on children's ages. In a recent survey of parents of children 2–17 years of age, the younger the child, the less the willingness of the parents to vaccinate immediately (22). The KFF COVID-19 Vaccine Monitor (23) is a research project tracking the public's attitudes and experiences with COVID-19 vaccinations. Among surveyed parents of children 5–11 years of age, 27% said they would get their children vaccinated for COVID-19 right away, but 33% preferred a “wait and see” policy. A total of 5% said they would only vaccinate if required, and 30% stated they would definitely not vaccinate their children. Parents’ main concerns regarding vaccinating their younger children aged 5–11 included potential unknown long-term effects, serious side effects of the vaccine (including the child's future fertility), and concerns related to access or information-related barriers to vaccination (23).

We found that Israeli Arabs and ultra-orthodox Jews, two distinct Israeli minorities, had significantly lower vaccination rates than non-ultra-orthodox Jews. In addition, most of the children in these two groups received only a single dose of the COVID-19 vaccine. The multivariate analysis also identified that these two sectors were two independent demographic determinants associated with significantly low vaccination rates.

Vaccination is the most effective public health strategy and the most successful policy in preventing morbidity and mortality of infectious diseases. Hence, parental compliance with children's vaccination is an essential tool for achieving high vaccination coverage and reducing the prevalence of diseases (24). Ali-Saleh et al. (25) conducted a cross-sectional survey among 2,483 Arab parents in Israel to assess the factors associated with their decision to vaccinate their children with COVID-19. Intention to vaccinate the children was positively and highly related to attitudes toward vaccination, subjective norms, and effectiveness of the vaccination. Weaker, yet positive significant correlations, were noted between the intention to vaccinate the children and parents' perception of susceptibility and severity of the disease. Multiple logistic regression was used to assess the extent to which the study determinants were related to the intention to vaccinate the children against COVID-19. Older parents and parents who were themselves vaccinated showed higher odds for positive intention to vaccinate their children. In addition, higher positive attitudes toward vaccination, higher supportive subjective norms, higher perceived susceptibility to the virus, lower barriers to vaccination, and greater perceived effectiveness of the vaccination – were related to higher odds of intention to vaccinate the children (25). There were also linguistic, technical, perceptual, cultural, and religious barriers against vaccination in the Arab population (26, 27).

Several studies counted multiple variables for the lower uptake of the COVID-19 vaccine amongst ultra-orthodox Jews and Arabs in Israel. These variables included hesitancy or lack of vaccine confidence, concerns about known/possible side effects and vaccine safety, concerns about adverse effects on fertility and pregnancy, limited exposure to the media, mistrust of authorities, language and communication barriers, need for child care during vaccination, and insufficient transportation to vaccination sites (26, 28, 29).

Similar logistical, perceptual/behavioral, and cultural impediments to vaccination that exist in both the ultra-orthodox Jewish and Arab populations may be a factor in the low vaccination rates in these sectors (27, 30). We suggest applying targeted interventions specifically appropriate for these sectors in order to increase COVID-19 vaccination rates in these groups. These interventions should include the following: 1. building public trust through culturally appropriate messages in the digital media (including newspapers, recorded phone messages, and billboard posters), transparency regarding vaccine efficacy and safety data, and role-modeling by political/religious/healthcare opinion leaders; 2. easing access to vaccination sites by using mobile vaccination units serving members placed in cities and in villages; 3. establishment of dedicated task forces and appointment of vaccine coordinators for Arab and ultra-Orthodox populations; 4. establishment of the Green Pass program for traveling abroad and other incentives.

Children with high SES had a high COVID-19 vaccination rate of 50.5%, which was significant in both the univariate analysis and multivariate analyses. A recent systematic review and meta-analysis to determine the acceptance rate of the COVID-19 vaccine and the related factors showed that the COVID-19 acceptance rate was higher among individuals with high SES (31).

Regarding the household size, we showed that the highest vaccination rate was among families of four households and the lowest among families that included six persons or more. The families of the Israeli Arab and ultra-orthodox Jews were in general from lower SES with high fertility rates (18, 19).

In our study, children who were vaccinated with the influenza vaccine had a higher rate of COVID-19 vaccination, which reflects a tendency of vaccine acceptance. Indeed, a cross-sectional study that investigated the medical factors which are associated with caregivers' intention to vaccinate their children against COVID-19 in 513 families, showed that children of caregivers who were unsure or did not intend to vaccinate their children against COVID-19 had a lower percentage of seasonal influenza vaccinations over the past five years in comparison to children of caregivers who intended to vaccinate their children against COVID-19 (60% vs. 81%) (32).

As mentioned earlier, children with various underlying medical conditions demonstrated relatively high vaccination rates. The highest second COVID-19 vaccine dose uptake was among children with type 1 diabetes mellitus, heart failure, treatment with biological agents, asthma, and obesity. Obesity and diabetes are known risk factors for COVID-19-associated severe morbidity and mortality among adults (33, 34). Woodruff et al. noted that of 2,293 pediatric hospitalizations in the United States, approximately 30% had severe COVID-19 illness. They observed that several comorbidities, such as obesity, diabetes, neurologic and cardiovascular diseases, were risk factors for severe COVID-19 (35). This was also true in a similar study that showed children with comorbidities such as obesity, diabetes, cardiovascular disease, chronic lung diseases other than asthma, seizure disorders, or immunocompromised had a high prevalence of severe COVID-19. Conversely, asthma, neurodevelopmental delay, and complex genetic disorders, such as Down's syndrome, had no effect on the risk of severe COVID-19 (10). We postulate that defining the high-risk groups for severe COVID-19 illness may be an important tool for increasing the awareness and priority for vaccination against SARS-CoV-2. We showed similar results in two recent studies regarding COVID-19 vaccine compliance in both Israeli adolescents and adults (36, 37).

The secondary aim of our study was to explore the infection rates among vaccinated and unvaccinated COVID-19 children. Lower rates of confirmed SARS-CoV-2 positivity by PCR were detected in the vaccinated children compared to unvaccinated ones and in children who received two vaccine doses, compared to a single vaccine dose. On November 26, 2021, the WHO declared the emerging SARS-CoV-2 Omicron variant (Pango Lineage B.1.1.529) as a variant of concern (38). Our study period from November 21st, 2021 to April 30th was mostly within the Omicron period. The Omicron variant is considered highly transmissible, spreading at a much faster rate than the other SARS-CoV-2 variants (39). It is able to evade immunity from previous vaccines or infections more extensively than previous variants, making existing vaccines less effective against the variant (40). This explains the relatively high rates of confirmed SARS-CoV-2 positivity by PCR which were detected in the vaccinated children. No death was recorded among children who received the COVID-19 vaccine.

The vaccination rate among families in which both parents were unvaccinated against COVID-19 was minimal, compared to the vaccination rate in children of families in which one of the parents was vaccinated against COVID-19 and of families in which both parents had COVID-19 vaccination.

Hayek et al. (41) evaluated the protective effect of parental COVID-19 vaccination provided to children. They studied two periods separately- an early period (Alpha variant), and a late period (Delta variant). They found that when a single parent was vaccinated, there was a 26.0% and a 20.8% decreased risk of infection of their children in the early and late periods, respectively; and when two parents were vaccinated, there was a decreased risk (71.7% and 58.1%, respectively). The authors concluded, that parental vaccination provides substantial protection for unvaccinated children in the household.

In a similar study, Prunas et al. (42) examined the effectiveness of vaccination with the BNT162b2 Pfizer-BioNTech vaccine against household transmission of SARS-CoV-2 in Israel before and after the emergence of the B.1.617.2 (Delta) variant. They found that vaccination reduced susceptibility to infection by 89.4%, whereas vaccine effectiveness against infectiousness was 23.0% during 10–90 days after the second dose. Total vaccine effectiveness was 91.8%.

In a study estimating community-level protection resulting from vaccinated individuals, Milman et al. found that the rates of vaccination in each of the 177 Israeli geographical areas were associated with a substantial later decline in infections among a cohort of individuals younger than 16 years, who were unvaccinated. They also showed that, on average, for every absolute 20% increase in the number of vaccinated individuals, the positive test fraction of the unvaccinated population decreased by a factor of ∼2 (43).

Layan et al. (44) described the impact of BNT162b2 vaccination and isolation on SARS-CoV-2 transmission on Israeli healthcare workers of a single central tertiary medical center and their family members. A total of 269 of 687 (39%) household contacts developed SARS-CoV-2 infection. Of those, 170 (63%) developed symptoms. Children below 12 years old were less susceptible than adults/teenagers. COVID-19 vaccination reduced the risk of infection among adults and teenagers. Isolation reduced the risk of infection of unvaccinated adults/teenagers [risk ratio (RR) = 0·11, 95% CI: 0·05–0·19] and child contacts (RR = 0·16, 95% CI: 0·07–0·31) compared to unvaccinated adults/teenagers who did not isolate. Infectivity was significantly reduced in vaccinated cases (RR = 0·22, 95% CI: 0·06–0·70). They concluded that vaccination reduces both the risk of infection and transmission if infected.

Yigit et al. (45) estimated the indirect protection of children *via* the COVID-19 vaccination of household members. A total of 2,289 (18.4%) of 12,442 patients aged <18 years were vaccinated, 91.4% with BNT162b2mRNA vaccine, and 8.6% with inactivated COVID-19 vaccine. SARS-CoV-2 infection was significantly lower in the vaccinated group (fully and incompletely) (*p* < 0.001). Unvaccinated and incompletely vaccinated had a higher risk of COVID-19 infection compared with fully vaccinated patients. However, they found no significant association between the COVID-19 RT-PCR positivity rates of patients and the vaccination status or vaccine preferences of household members.

The main strength of our study relates to comprehensive pediatric population-based updated data of their demographics, child and parental vaccination status, and comorbidities. Our study has a few limitations. First, our data were based on a single district level and not on a nationwide level, and therefore might not necessarily be representative of the entire country. Approximately 74% of the Israeli population are Jews and 21% are Arabs, but in our study population 93.5% are Jews and 6.5% are Arabs, the study population is thus not fully representative of the Israeli population. Despite that, the various ethnic groups and socioeconomic levels are well represented in the district. Second, the relatively short follow-up duration did not enable conclusions to be drawn about the long-term effect of the COVID-19 vaccine, as well as the compliance to a vaccine booster dose. Third, we could not refer to unmeasured confounders factors (e.g., behavioral factors such as wearing masks, washing hands, close contact with persons with COVID-19 at school, or social distancing), that might have biased the results. Fourth, the determination of household membership was based on demographic data in our database; in some cases, it is possible that additional individuals (e.g., grandparents or nonparent caregivers) reside in the same household.

## Conclusion

The present study demonstrates that the COVID-19 vaccination rates among children aged 5–11 years were relatively low in minorities (ultra-orthodox Jews, Arabs), in households of six family members or more, and in families in which both parents were unvaccinated. High vaccine uptake was found in children with high SES and patients treated with biologic agents, patients who underwent solid tumor transplantation, and patients with type 1 diabetes mellitus, asthma, and ADHD. It is essential to address determinants associated with parents' hesitancy to increase vaccination rates of children.

## Data Availability

The data analyzed in this study is subject to the following licenses/restrictions: Medical confidentiality. Requests to access these datasets should be directed to shine6@walla.co.il.
